# Response of Fungal Communities to Anthropogenic Disturbances in a Tropical Forest

**DOI:** 10.1002/pei3.70164

**Published:** 2026-05-16

**Authors:** Julius Leiririo, Hannah Karuri, Joshua Muli, Mileen Kawira

**Affiliations:** ^1^ Department of Biological Sciences University of Embu Embu Kenya; ^2^ Department of Physical Sciences University of Embu Embu Kenya

**Keywords:** diversity, fungi, land use change, protected area, soil biodiversity

## Abstract

Conversion of forests to agricultural land leads to a decline in soil biodiversity and it affects the ecosystem services that they provide. The rising population around Mt. Kenya forest and an increase in demand for land and forest resources has led to deforestation. However, the impact on fungal communities remains unknown. High‐throughput sequencing was used to assess fungal diversity in the protected part of the Mt. Kenya forest, which is predominantly woodland, and in unprotected areas dominated by shrubland. The most significantly (*p* < 0.05) abundant fungal genera in the woodland were *Mortierella* (23.3%) and *Metarhizium* (5.4%). *Fusarium* (8.5%) and *Aspergillus* (3.5%) were significantly higher in the shrubland. An evaluation of the primary lifestyle showed a significantly (*p* < 0.05) higher number of animal parasites (16.3%) and soil saprotrophs (46.2%) in the woodland. Conversely, shrubland had a higher abundance of plant pathogens (20%). Beta diversity analysis showed a clear separation of fungal communities in the woodland and shrubland. Redundancy analysis showed a positive correlation of *Mortierella* with Mn and C. *Metarhizium* and *Aspergillus* were negatively correlated with clay and C, respectively. These results provide important information for the conservation of fungal communities in the Mt. Kenya forest and it will also inform management decisions aimed at improving soil health.

## 1 Introduction

The global land surface is covered by 30% forest cover, which holds 45% of carbon (C) stocks. Forests sequester carbon dioxide (CO_2_) emissions contributing to climate change mitigation. In particular, tropical forests sequester a substantial amount of carbon and they account for approximately 25% of the terrestrial C (Bonan 2008). Forests undergo natural and anthropogenic disturbances across a range of spatial and temporal scales (Ratajczak et al. 2018). In Africa, the conversion of forests to cropland is a major driver of forest decline (Masolele et al. 2024). These disturbances affect the distribution, abundance, and diversity of above and below ground organisms, including fungal communities, which can lead to negative effects on forest health (Gil‐Fernández et al. 2025; Sapsford et al. 2021; Shi et al. 2019).

Ectomycorrhizal (ECM) and arbuscular mycorrhizal fungi (AMF) form symbiotic relationships with tree species and they play critical roles in nutrient cycling (Phillips et al. 2013), tree nutrient acquisition (Smith and Read 2010), modulating metabolic activities of soil organisms, C sequestration (Steidinger et al. 2019) and shaping plant community diversity (Chen et al. 2019). In particular, ECM form critical symbiotic relationships with trees which depend on the fungi for water and nutrient acquisition (Smith and Read 2010). These fungi are sensitive to disturbances and their re‐establishment after disturbance can take several years (Spake et al. 2016). A reduction in species richness and changes in the community composition of ECM fungi was observed in a forest undergoing disturbance through large clear cuts (Rianhard et al. 2025). Transformation of a tropical forest into rubber/oil plantation resulted in low abundance of ECM fungal species (Brinkmann et al. 2019). In Cameroon, tree harvesting and slash and burn agricultural practices caused an almost complete loss of ECM (Onguene 2022). Disturbance has also been shown to affect the abundance and diversity of AMF in forests. For instance, conversion of forest to farmland reduces AMF diversity (Palta et al. 2025). Similarly, anthropogenic disturbances negatively affect AMF abundance and diversity in a forest after disturbances (Sapsford et al. 2021). However, a different study suggests that disturbance can equalize AMF diversity (Garcia de Leon et al. 2018).

Globally, less than 10% of mycorrhizal biodiversity hotspots are located in protected areas highlighting an urgent need for coordinated conservation efforts to meet the Kunming–Montreal Global Biodiversity Framework goals (Van Nuland et al. 2025). Africa has a high number of ECM trees with fungi that have a wide variation in abundance and diversity (Corrales et al. 2018). The Mt. Kenya forest ecosystem is a hotspot of biodiversity and part of it is a world heritage site that provides key ecosystem services (Kenya Wildlife Service 2010). The increase in demand for wood products coupled with climate change has led to large‐scale disturbances in the unprotected areas of Mt. Kenya forest (Rotich et al. 2025). These natural and anthropogenic pressures are a threat to forest health (Millar and Stephenson 2015), biodiversity, and lead to reduced ecosystem productivity and resilience (Pörtner et al. 2023). They also affect the abundance and community composition of fungi (Corrales et al. 2018).

Thus, understanding the effect of disturbances on fungi in forest ecosystems is necessary in order to protect the biodiversity and the ecosystem services provided by the forest. In this study, we investigated the fungal diversity in protected and unprotected areas of Mt. Kenya forest. We hypothesized that protected areas of the forest would have a high abundance and diversity of fungi. This study provides baseline information that can be used to inform forest conservation strategies and guide restoration planning.

## 2 Materials and Methods

### 2.1 Study Sites and Sample Collection

Soil samples were collected in September 2023 from the protected (Irangi forest) and unprotected (Njukiri forest) parts of Mt. Kenya forest, Kenya. Samples were collected from 15 sites in Irangi forest (0°20′45.1″S 37°28′51.3″ E), a protected site with minimal disturbances, predominantly consisting of woodland. Additionally, 15 sites were sampled in the unprotected Njukiri forest (0°30′36.7″S 37°27′30.0″ E), an area experiencing anthropogenic disturbances and predominantly covered by shrubland. The requisite permits were obtained from the Kenya Forest Service. From each site, a 5 cm x 10 cm auger was used to collect soil samples from a 30 m x 30 m area marked with two transects. The distance between sites was 1 km. A composite sample from each site was placed in a plastic bag and transported to the laboratory in a cool box. A sub‐sample of the soil was stored at −20°C for DNA extraction; while the rest was sent for analysis of soil properties at Kenya Agricultural and Livestock Research Organization, National Agricultural Research Laboratories. Mehlich et al. (1962) method was used to determine the amount of Magnesium (Mg), Manganese (Mn), Potassium (K), Calcium (Ca), Sodium (Na), Phosphorous (P), and pH. Levels of C (Anderson and Ingram 1994) and Nitrogen (N) (Bremner and Mulvaney 1982) were determined through colorimetric and Kjeldahl digestion techniques, respectively. Iron (Fe), Copper (Cu), and Zinc (Zn) were assessed using Atomic Absorption Spectrophotometer; while soil texture was examined using the hydrometer technique (Klute 1986). According to the Kenya Meteorological Department, the average monthly temperature and rainfall at the sampling sites were 21.5°C and 39.27 mm, respectively.

### 2.2 Extraction of DNA and Sequencing

The DNeasy Power Soil Pro Kit (Qiagen) was used to extract DNA from each sample (250 mg) following manufacturer's instructions. Blank tubes served as negative controls. The DNA was kept at −80°C before subsequent analysis. The ITS2 region was amplified using the ITS3 ((Forward): 5′‐GCATCGATGAAGAACGCAGC‐3′/ITS4 (Reverse): 5′‐TCCTCCGCTTATTGATATGC‐3′) primer set with the PCR conditions described by Op De Beeck et al. (2014). Briefly, initial denaturation for 2 min at 95°C, 40 cycles of denaturation for 30 s at 95°C, annealing for 30 s at 55°C, extension at 72°C for 1 min and a final extension for 10 min at 72°C.

The amplicons were visualized on 2% agarose gel and purified using Ampure XP beads before library preparation and sequencing at Scripps Research Institute (San Diego, CA, USA). Paired‐end sequencing was carried out on NextSeq 2000 (2 × 250bp) (Illumina Inc., CA, USA).

### 2.3 Bioinformatics and Statistical Analyses

Fungi sequences were quality filtered, demultiplexed, and chimeras removed using the Lotus2 pipeline (Özkurt et al. 2022). The processed OTUs were assigned to genus and phylum using vsearch against the UNITE reference database (Nilsson et al. 2019). Further analysis of the resulting phyloseq object was carried out in R software. Before downstream analysis in R, singletons and doubletons were removed. The FungalTraits database (Põlme et al. 2020) was used to determine the ecological guild of the fungal genera based on the primary life style. The raw sequence data is deposited in the Sequence Read Archive under BioProject number PRJNA1186071.

All statistical analyses were conducted in R studio v 4.2.3. Data that was not normally distributed was standardized using Shapiro–Wilk and Levene's tests to meet normality and homoscedasticity assumptions, respectively. In case of violation of the assumptions, the Kruskal‐Wallis test was used and the *p* values were reported after Benjamini‐Hochberg correction. A comparison of alpha diversity of fungi in the two ecosystems was done by computing the abundance‐based coverage estimator (ACE), Fisher and Shannon diversity indices on rarefied sequence reads. Beta diversity in the forest ecosystems was analyzed using permutational multivariate analysis of variance (PERMANOVA, nperm = 9999) and the group dispersions were determined through Permutational Analysis of Multivariate Dispersion (PERMDISP). A principal coordinate analysis (PCoA) was used to visualize ordinations of the fungi based on a Bray‐Curtis distance matrix. An analysis of variance (ANOVA) was used to determine the differences in soil properties across the forest ecosystems. Variance inflation factor (VIF) analysis was used to filter collinear variables (VIF > 10). Relationships of the fungal communities with soil physico‐chemical properties were determined using redundancy analysis (RDA). Significance of the RDA model was assessed using permutation‐based ANOVA (nperm = 999).

## 3 Results

**FIGURE 1:**
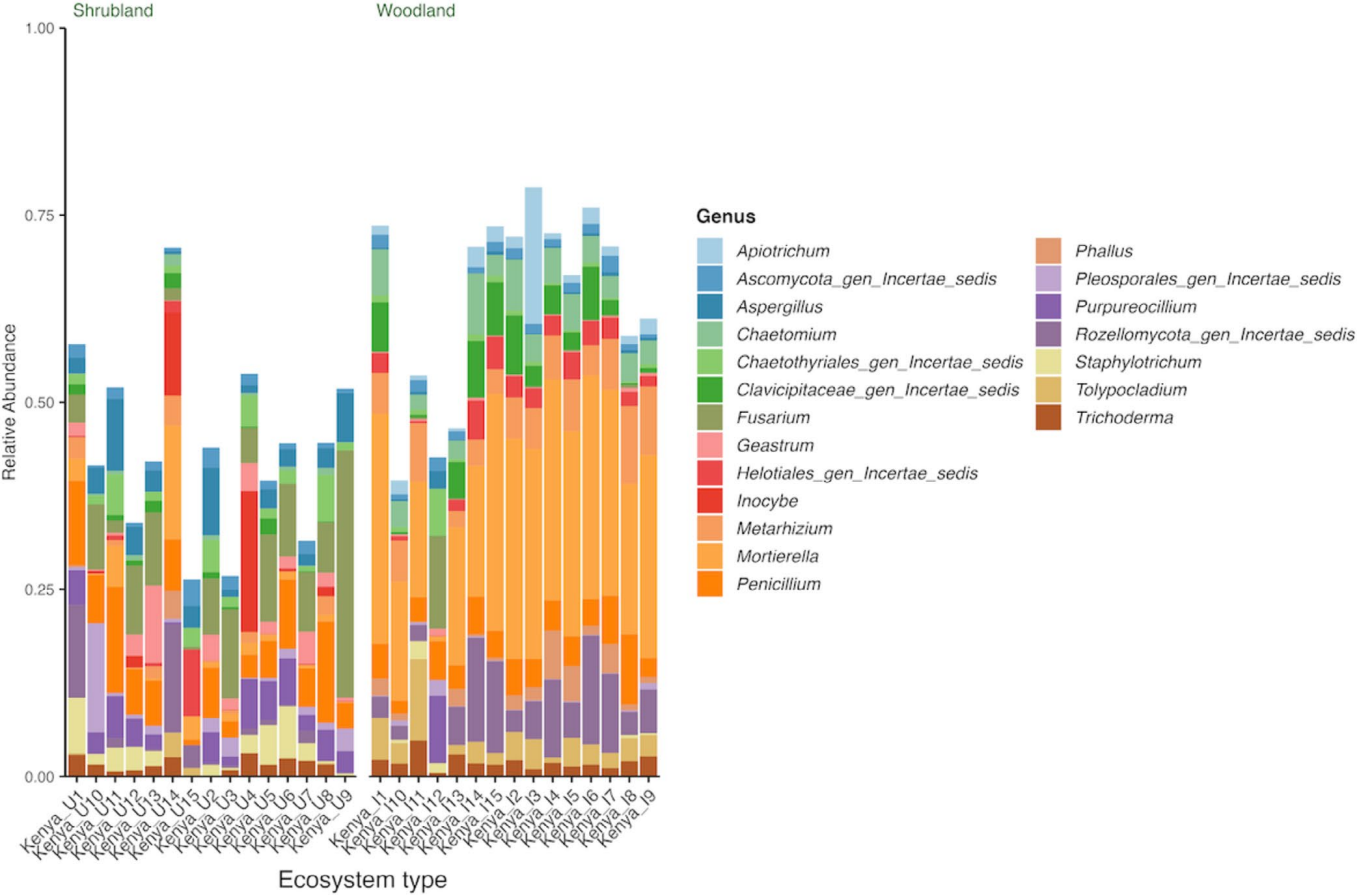
Top 20 fungal genera in unprotected shrubland and protected woodland areas of Mt. Kenya forest.

**FIGURE 2:**
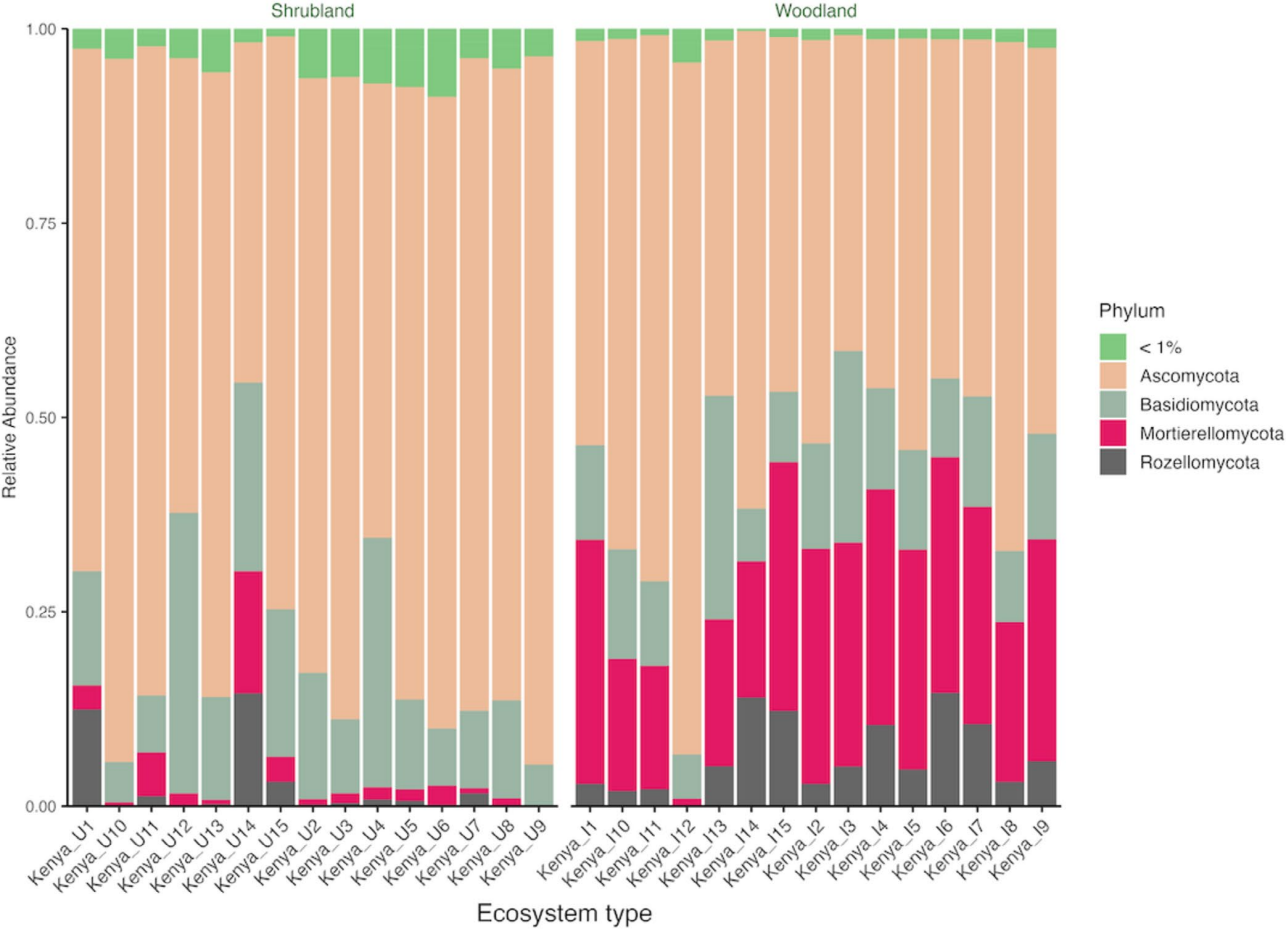
Relative abundance of fungal phyla in unprotected shrubland and protected woodland areas of Mt. Kenya forest.

The total number of reads from the two systems was 5,357,490 with an average of 178,583 per sample. Shrubland and woodland had 2,594,219 and 2,763,271 reads, respectively. A comparison of the top 20 genera in the two ecosystems showed that the most significantly (*p* < 0.05) abundant genera in woodland were *Mortierella* (23.3%) and *Metarhizium* (5.4%). *Fusarium* (8.5%) and *Aspergillus* (3.5%) were significantly higher in the shrubland (Figure 1). Ascomycota (55%) and Mortierellomycota (23.9%) were the most abundant phyla in woodland, while shrubland was dominated by Ascomycota (75.4%) (Figure 2). An evaluation of the primary lifestyle (Põlme et al. 2020) showed a significantly (*p* < 0.05) higher number of animal parasites (16.3%) and soil saprotrophs (46.2%) in the woodland. Conversely, shrubland had a higher abundance of plant pathogens (20%).

**FIGURE 3:**
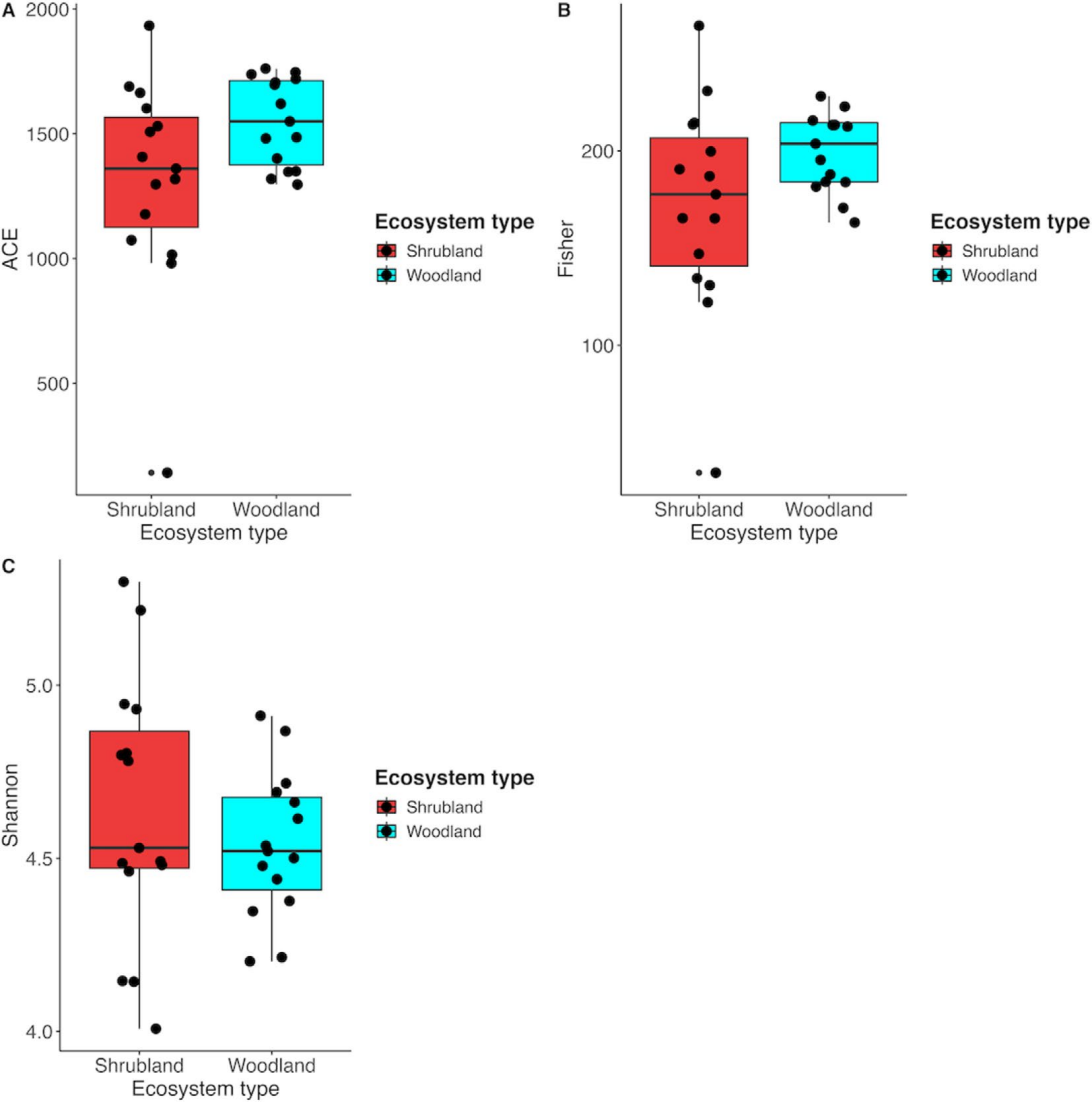
Shannon, Fisher, and abundance‐based coverage estimator (ACE) of fungi in unprotected shrubland and protected woodland areas of Mt. Kenya forest.

**FIGURE 4:**
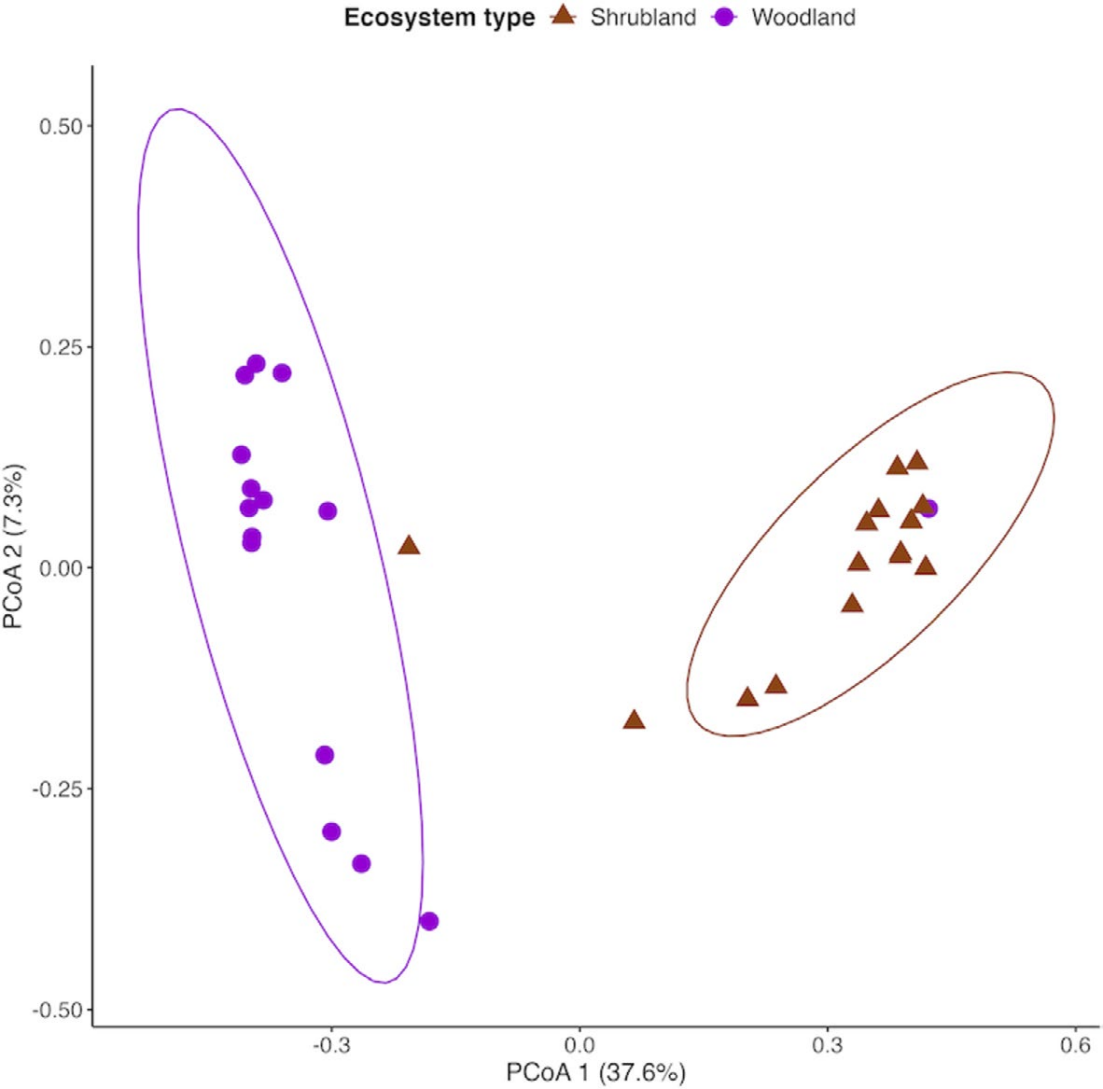
Principal coordinate analysis of beta diversity of fungi in unprotected shrubland and protected woodland areas of Mt. Kenya forest.

An analysis of alpha diversity metrics in the two ecosystems showed significant differences (*p* < 0.05) in ACE diversity but no differences in Fisher and Shannon diversity indices (Figure 3). The beta diversity analysis showed a distinct separation of fungi OTUs in the two forest ecosystems (PERMANOVA, *p* < 0.001). The first and second PCoA axes accounted for 44.9% of the total variance (Figure 4).

**FIGURE 5:**
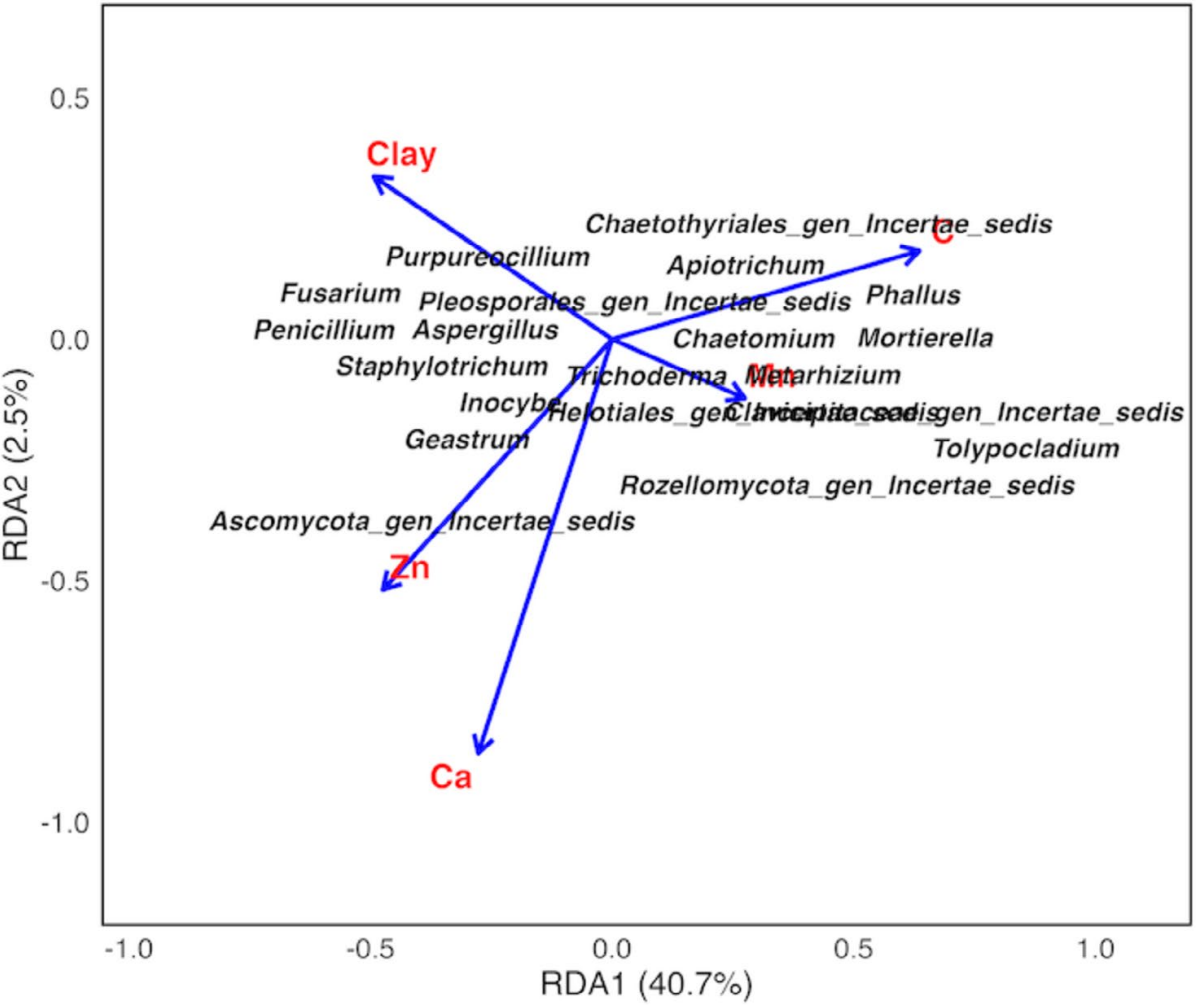
Redundancy analysis of fungal communities and selected soil properties in unprotected shrubland and protected woodland areas of Mt. Kenya forest.

There were significant differences in all the soil variables, except for Mn and silt (Table 1). The RDA analysis showed a positive correlation (*p* = 0.001) of *Mortierella* with Mn and C. *Metarhizium* and *Aspergillus* were negatively correlated with clay and C, respectively. *Fusarium* was positively correlated with clay and negatively with C and Ca (Figure 5). The first and second RDA axes represented 40.7% and 2.5% of the variance, respectively.

**TABLE 1:** Soil physical and chemical properties (Mean ± Standard Error) of samples collected from unprotected shrubland and protected woodland areas of Mt. Kenya forest.

	Shrubland		Woodland	
	Mean	SE	Mean	SE
pH	5.6	0.22	3.9	0.07
Total Nitrogen %	0.3	0.02	0.6	0.06
Total organic Carbon %	2.9	0.20	5.8	0.63
Phosphorous ppm	33.2	3.15	22.2	2.90
Potassium meq %	0.7	0.06	0.4	0.02
Calcium meq %	14.5	5.45	0.3	0.02
Magnesium meq %	3.7	0.33	1.7	0.15
Manganese meq %	0.6	0.02	0.6	0.11
Copper ppm	6.1	0.29	3.2	0.14
Iron ppm	35.6	8.32	72.5	6.38
Zinc ppm	27.5	4.75	11.5	0.73
Sodium meq %	0.2	0.02	0.2	0.01
Sand	17.1	0.47	36.3	4.47
Clay	70.8	1.47	50.4	4.43
Silt	12.1	1.16	13.3	0.57

## 4 Discussion

In forest ecosystems, fungi are a vital part of the biodiversity due to their central role in ecosystem functions. Disturbances may cause shifts in fungal communities, potentially affecting forest health. In this study, *Mortierella* was significantly higher in the woodland compared to the shrubland. Most *Mortierella* species are endophytes or saprophytes, with some being reported as pathogenic (Crowther et al. 2012; Vélez et al. 2020). High abundance of *Mortierella* species in the woodland may be attributed to the availability of more nutrients and high levels of decomposition compared to the shrubland. *Mortierella* species are considered r‐selected and they grow rapidly on organic matter, and its abundance increases under high nutrient conditions (Schmidt et al. 2008). It increases the uptake of P by plants through its phosphate‐solubilizing properties (Osorio and Habte 2013). This genus has been shown to serve as a biological control agent of nematodes (DiLegge et al. 2019) and pathogens that cause decline in tree species (Diez‐Hermano et al. 2024). On the other hand, the entomopathogenic fungi, *Metarhizium*, has been reported in undisturbed forests similar to the observation in this study (Riguetti Zanardo Botelho et al. 2019). It is usually found in close association with insects (Deaver et al. 2019; J. Zheng et al. 2024), and it is known to promote plant growth through suppression of pathogenic fungi (Leger 2008). The high abundance of *Metarhizium* in the protected woodland may be attributed to low levels of disturbance, the high availability of insect hosts and different plant species, and the modification of soil structure by litter, which creates a conducive environment for proliferation of the fungi (Jaronski 2010; Klingen et al. 2002; C. Wang and St Leger 2007).

The unprotected shrubland had a higher relative abundance of *Fusarium*, similar to observations from other studies (Cabello and Arambarri 2002; Shi et al. 2019). This plant‐pathogenic fungi causes seedling diseases and pitch canker in coniferous trees (Gordon et al. 2015; Landeras et al. 2005) and “fusarium wilt” in some tree species (Mao et al. 2021; Zhao et al. 2020). In addition, in this study, it was observed that the shrubland had a higher abundance of Ascomycota compared to the protected woodland, which is consistent with other studies (Iacono et al. 2025; Koutika 2025; Liu et al. 2022). This may be due to the fact that the substrates in the woodland have recalcitrant material with high lignin content in comparison with the labile materials in the shrubland, which are a preferred nutrient source for Ascomycota (Voříšková and Baldrian 2013). In addition, Ascomycota can withstand high levels of stress (Egidi et al. 2019).

Disturbance in the shrubland may have contributed to the increased abundance of plant pathogens that was observed in this study as previously reported (Brinkmann et al. 2019; Fang et al. 2023; Shi et al. 2019). Further, Shi et al. (2019) propose that the other possible causes of increased pathogenic fungi may include changes in the microclimatic conditions of the shrubland, the lack of competition from saprophytic fungi, and reduced tree cover that promote the spread of pathogenic fungal spores. Conversely, the high abundance of soil saprotrophs that we observed in the woodland may be due to high availability of substrates from different tree species at various decomposition stages (Pietras et al. 2025), the influence of soil properties, or the diversity of trees in the woodland. Soil saprotrophs are positively correlated to high levels of organic matter and N (Fang et al. 2023), and they play a key role in decomposition, which increases C and promotes plant growth (Talbot et al. 2013). Shi et al. (2019) also observed a low abundance of saprophytic fungi in disturbed areas, and they attributed this to increased disturbance, high levels of soil P, or alkaline soils.

Alpha and beta diversities are also influenced by forest disturbance with natural forests having higher diversity (Brinkmann et al. 2019; Song et al. 2019). Similar to observations made by Osburn et al. (2019), disturbance did not affect the Shannon diversity of fungi in the current study, likely due to the influence of plant‐related (Osburn et al. 2019) or environmental factors (Mollier et al. 2024) not examined here. However, as reported by Modi et al. (2020) we observed a distinct differentiation in beta diversity of fungal communities. Loss of fungal species, change in vegetation type and soil pH in the disturbed forest may be a possible reason for the differences in beta diversity (Lan et al. 2021; Lladó et al. 2018; Sawada et al. 2021).

In addition to disturbance, other factors, such as tree species, climate, and soil properties affect fungal diversity in forests (Fang et al. 2023; Pietras et al. 2025). Land use intensity affects the soil physico‐chemical properties which in turn affect the fungal diversity (Fang et al. 2023). The present study showed a significant influence of soil properties on fungal abundance with the first and second RDA axes representing 40.7% and 2.5% of the constrained variation, respectively. The relatively high contribution of the first axis indicates that soil properties accounted for a substantial portion of the variation in the abundance of fungal genera. In contrast, the low contribution of the second axis suggests that additional soil variables explained a small portion of the variation, with other factors such as disturbances or unmeasured environmental variables contributing to the remaining variation (Shi et al. 2019; Wang et al. 2014). A study on fungal communities in a sub‐tropical forest showed that *Mortierella* species are found within specific soil horizons (Qu et al. 2021), and they show a positive correlation with C (Luo et al. 2021) as per our observations. The distribution and abundance of *Metarhizium* is also influenced by soil properties. Contrary to our results, P, Cu, and pH were cited as the main soil properties influencing *Metarhizium* in forest soil (J. Zheng et al. 2024). Similarly, *Aspergillus* in a tropical forest showed a negative correlation with K and Fe (Solanki et al. 2024), which contrasts with our observations. Conversion of forests into agricultural land affects soil properties, which leads to a high abundance of *Fusarium* and a subsequent increase in N_2_O emissions (N. Zheng et al. 2020). In this study, the negative correlation between C and Ca, and the abundance of *Fusarium* is contrary to the reports by Maina et al. (2009). Apart from the factors listed above, seasons and climate also play a critical role in shaping fungal diversity (Castaño et al. 2018; Shigyo et al. 2019; Voříšková et al. 2014; M. Wang et al. 2014). These aspects, which we acknowledge as limitations of the study, were not assessed but they can be addressed in further studies.

## 5 Conclusion

Our results demonstrate that unprotected and protected areas of Mt. Kenya forest have distinct fungal diversity. The disturbed parts of the forest also exhibit a higher abundance of pathogenic fungi, which may pose risks to forest health. These findings provide a valuable basis for informing forest conservation policies and legislation that protect biodiversity, while contributing to the goals of the Kunming–Montreal Global Biodiversity Framework. They further support the development of forest management strategies that prioritize the conservation of fungal diversity. Overall, this highlights the need for land use policies that effectively balance agricultural demands with the conservation of forest biodiversity and the ecosystem services it provides.

## Funding

This work was supported by the Society for the Protection of Underground Networks.

## Conflicts of Interest

The authors declare no conflicts of interest.

## Data Availability

The sequencing data are available in National Center for Biotechnology Information at https://www.ncbi.nlm.nih.gov/, BioProject ID PRJNA1186071. On reasonable request, the corresponding authors will provide any other datasets analyzed during the current work.
